# Mass Azithromycin Distributions and Childhood Clinic Visits in Niger: A Community-Randomized Trial

**DOI:** 10.4269/ajtmh.25-0462

**Published:** 2025-10-23

**Authors:** Zijun Liu, Renee F. N. Casentini, Ahmed M. Arzika, Abdou Amza, Ramatou Maliki, Catherine Cook, Carolyn Brandt, Ritesh Tiwari, Kieran S. O’Brien, Elodie Lebas, Travis C. Porco, Jeremy D. Keenan, Thomas M. Lietman

**Affiliations:** ^1^ Francis I. Proctor Foundation, University of California San Francisco, San Francisco, California;; ^2^ The Carter Center, Niamey, Niger;; ^3^ Centre de Recherche et Interventions en Santé Publique, Birni N’Gaoure, Niger;; ^4^ Programme Nationale de Santé Oculaire, Niamey, Niger;; ^5^ Universite Abdou Moumouni de Niamey, Programme FSS, Niamey, Niger;; ^6^ DataSlexIndia, New Dehli, India

## Abstract

The community-randomized, placebo-controlled *Macrolides Oraux pour Réduire les Décès avec un Oeil sur la Résistance* (MORDOR) trial revealed that biannual mass azithromycin distributions to preschool children reduced child mortality in sub-Saharan Africa. Other benefits may also be possible. Surveillance of local health post records was assessed from the Niger study site from 2014 to 2016. Community-level data on all clinic visits, diagnoses, and antibiotic prescriptions recorded in the health post logbooks were retrospectively extracted for the period spanning from 12 months before baseline to 12 months after baseline. During the 12-month period after the communities were randomized in the MORDOR trial, a total of 5,229 clinic visits were documented in the azithromycin group, compared with 7,647 in the placebo group (incidence rate ratio [IRR]: 0.82; 95% CI: 0.79–0.85); 2,011 malaria diagnoses were documented in the azithromycin group, compared with 3,366 in the placebo group (IRR: 0.71; 95% CI: 0.46–1.39); and 2,218 courses of antibiotics were documented in the azithromycin group, compared with 3,297 in the placebo group (IRR: 0.81; 95% CI: 0.77–0.85). The reduction in clinic visits and clinic-prescribed antibiotics is consistent with a reduction in infectious diseases in the azithromycin-treated communities.

## INTRODUCTION

The *Macrolides Oraux pour Réduire les Décès avec un Oeil sur la Résistance* (MORDOR) trial revealed that childhood mortality among children aged 1–59 months in Malawi, Niger, and Tanzania was ∼14% lower in communities randomly assigned to receive biannual doses of azithromycin, compared with communities that received a placebo.^1^ The Community Health with Azithromycin Trial (2019–2023) and the *Azithromycine pour la Vie des Enfants au Niger: Implementation et Recherche* (2020–2023) trial have subsequently confirmed the MORDOR trial’s findings, with an 18% and 14% reduction in childhood mortality, respectively.^2^^,^^3^ Verbal autopsies were performed in the communities in Niger that were involved in the MORDOR trial to determine the cause of death in the azithromycin and placebo arms.^4^ The results suggest that azithromycin reduces child mortality due to malaria, dysentery, pneumonia, and meningitis.^4^

Health post visits (termed clinic visits) were analyzed at the Niger site of the MORDOR trial, before and after the first distributions of azithromycin and placebo. Primary care in rural communities is provided by government-supported Centres de Santé Intégrées (referred to as health posts in the present study), which offer free healthcare to preschool children. Clinic visits, specific disease diagnoses (malaria, respiratory, and gastrointestinal), and antibiotic prescriptions were compared between communities randomly assigned to receive a placebo and those randomly assigned to receive azithromycin.

## MATERIALS AND METHODS

### Eligibility criteria.

Communities with populations between 200 and 2,000 inhabitants were eligible for enrollment but remained in the trial even when their numbers fell outside this range. Children were enrolled in the study if they were between 1 and 59 months of age and weighed at least 3,800 g.

### Data collection.

The data for the present study were obtained from a subset of communities located at the Niger site of the MORDOR trial, which was funded by the Bill & Melinda Gates Foundation (MORDOR ClinicalTrials.gov number: NCT02048007). Detailed methods, collection protocol, and primary outcomes have been reported previously.^1^ Briefly, communities in Niger, Malawi, and Tanzania were randomly assigned to receive either mass azithromycin (at a dose of 20 mg/kg) or an identical-appearing placebo every 6 months for all children aged 1–59 months, distributed during door-to-door visits. The primary outcome of the trial was mortality.^1^ The Niger site of the MORDOR trial enrolled *grappes* (i.e., government-defined health units; referred to as communities in the present study) with 200–2,000 inhabitants on the most recent government census from two contiguous departments: Boboye and Loga. In this ancillary study, surveillance of health post records for all children aged 1 month to 5 years was performed retrospectively for a subset of health posts in the Loga Department. The health post records included communities that were ineligible for the MORDOR trial; however, only those that were eligible were included in the present study’s analysis. Data were collected from the routine logbooks kept at each health post and included the date of the visit, diagnosis, and treatment. Clinic staff diagnosed children on the basis of standardized WHO criteria for health facilities, Integrated Management of Childhood Illness.^5^^,^^6^ Rapid diagnostic tests were used to confirm malaria diagnoses. The number of clinic visits per community (the unit of randomization) in the 12-month interval after the first MORDOR distribution (from August 2015 to August 2016) was modeled in a negative binomial regression model that included the treatment arm and the log number of clinic visits in the 12-month interval before the first MORDOR distribution (January 2014 to January 2015) as predictors (Supplemental Figure 1).^7^^,^^8^ Azithromycin treatments were ongoing for ∼1 year after the present study.

### Subgroup analyses.

Similar analyses were performed for community-level counts of visits for malaria, respiratory infections (i.e., respiratory infection, pharyngitis, pneumonia, rhinitis, sinusitis, cough, common environmental-related cough, and chronic cough), gastrointestinal infections (i.e., digestive infection, diarrhea, diphtheria, dysentery, gastritis, intestinal infection, intestinal parasitosis, gastrointestinal functional disorders, and typhoid), and courses of antibiotics prescribed.

### Power calculation.

An estimated 187 communities in the Loga Department enrolled in the MORDOR trial provided 80% power to detect a 0.27 standard deviation unit reduction (Cohen’s *d*) in all-cause clinic visits in azithromycin-treated communities compared with placebo-treated communities. The power calculations were performed before data collection and are outlined in the statistical analysis plan for MORDOR extensions. A correlation coefficient of 0.89 was assumed between the number of clinic visits in the year preceding the intervention and the number of clinic cases after the intervention.

### Data entry.

Data were routinely recorded at the health posts on paper logbooks. Study data were collected and managed using REDCap (Vanderbilt University, Nashville, TN) electronic data capture tools hosted at the University of California San Francisco.^9^^,^^10^ The study team extracted data from photographs of logbook pages into REDCap, using double data entry with adjudication for discrepancies. The reconciled data were used for analysis in the present study. Unreadable or unadjudicable logbook data were excluded from the analysis.

## RESULTS

A total of 20,563 clinic visits for children aged 1–59 months from 187 communities in the Loga Department were documented in the 12-month interval before the MORDOR trial (January 1, 2014 to January 1, 2015) and 12,876 in the 12-month interval after the first MORDOR distribution (August 1, 2015 to August 1, 2016). Approximately half of the children live in communities located more than 5 kilometers from the health post. Baseline characteristics are displayed in Table 1 by arm.

**Table 1:** Baseline community-level characteristics by treatment arm

Characteristic	Placebo (*n* = 99 communities)	Azithromycin (*n* = 88 communities)	Overall (*N* = 187 communities)
No. children per community, median (IQR)	119.0 (80.5–179.0)	124.0 (87.5–198.0)	121.0 (84.0–189.0)
Distance to CSI (km), median (IQR)	5.5 (3.3–7.9)	5.3 (3.5–7.0)	5.4 (3.3–7.4)
Clinic visits in 12 months before MORDOR per community, median (IQR)	25.0 (4.0–86.5)	23.0 (5.0–72.3)	25.0 (4.0–78.0)
Malaria visits, median (IQR)	5.0 (0.0–31.0)	2.0 (0.0–23.0)	2.0 (0.0–26.0)
Respiratory visits, median (IQR)	7.0 (2.0–23.0)	11.0 (2.8–25.3)	8.0 (2.0–25.0)
Gastrointestinal visits, median (IQR)	1.0 (0.0–4.0)	1.0 (0.0–4.3)	1.0 (0.0–4.0)
Clinic visits prescribed antibiotics per community, median (IQR)	11.0 (1.5–34.5)	15.5 (2.8–36.3)	13.0 (2.0–36.0)

CSI = Centres de Santé Intégrées; IQR = interquartile range; MORDOR = *Macrolides Oraux pour Réduire les Décès avec un Oeil sur la Résistance*.

Azithromycin was associated with an 18% reduction in clinic visits compared with the placebo (incidence rate ratio [IRR]: 0.82; 95% CI: 0.79–0.85), even after controlling for pre-MORDOR visits. In the subgroup analysis of each diagnosis, after controlling for all pre-MORDOR clinic visits, malaria was the only diagnosis that decreased significantly, with a 29% reduction in the azithromycin group (IRR: 0.71; 95% CI: 0.67–0.75). The respiratory and gastrointestinal diagnoses did not differ significantly between the arms. There were 19% fewer clinic visits during which antibiotics were prescribed in the azithromycin arm compared with the placebo arm (IRR: 0.81; 95% CI: 0.77–0.85; Table 2).

**Table 2:** Number of clinic visits from azithromycin-treated communities compared with the placebo-treated communities

Outcome	IRR* (CI)	*P*-Value
All diagnoses (no. of clinic visits)	0.82 (0.79–0.85)	<0.001
Malaria (no. of clinic visits)	0.71 (0.67–0.75)	<0.001
Respiratory (no. of clinic visits)	0.80 (0.46–1.39)	0.43
Gastrointestinal (no. of clinic visits)	1.05 (0.59–1.86)	0.86
Antibiotics (no. of antibiotic prescriptions)	0.81 (0.77–0.85)	<0.001

IRR = incidence rate ratio.

* An IRR value less than 1 implies fewer clinic visits in the azithromycin group.

## DISCUSSION

Communities randomly assigned to receive azithromycin had significantly fewer total clinic visits, fewer visits due to malaria, and fewer courses of antibiotics prescribed compared with communities randomly assigned to receive the placebo. Visits due to respiratory infections were less common in the azithromycin arm, but this difference was not statistically significant. Similarly, there was no meaningful difference in visits due to gastrointestinal infections between the two treatment arms.

The observed decrease in overall clinic visits is consistent with the beneficial effect on childhood mortality and morbidity associated with azithromycin in previous studies.^1^^–^^3^^,^^11^ Azithromycin is considered to exhibit modest antimalarial effects, consistent with the results of the present study, which revealed a reduction in malaria with azithromycin distribution.^12^^–^^15^ A decrease in overall clinic visits alone might suggest a decrease in clinic visits during which antibiotics are prescribed. In addition, azithromycin may have treated the very infections that would have brought a child to the clinic. Previous findings from longitudinal studies, which revealed significant reductions in diarrhea and respiratory infections with the use of azithromycin, could not be confirmed in the current study.^15^^,^^16^ One household-randomized study in Burkina Faso and Mali revealed that pairing azithromycin with seasonal malaria chemoprevention resulted in a lower frequency of gastrointestinal infections, upper respiratory tract infections, and non-malarial febrile illnesses compared with a placebo.^15^ Studies on changes in the gut microbiome after mass azithromycin distribution have revealed a reduction in pathogens known to cause diarrhea, including *Campylobacter* spp. and *Shigella *spp.^17^^,^^18^ Azithromycin has also been shown to reduce clinic visits for skin conditions (e.g., erysipelas) when used in combination with ivermectin. A longitudinal study focused on scabies and impetigo revealed that treating randomly selected sets of villages in the Solomon Islands with azithromycin and ivermectin resulted in fewer clinic visits and a decrease in skin sores, boils, and abscesses.^16^ Visits for skin infections were not recorded in the present study but are currently being collected and will be assessed in future studies. In the current study, azithromycin was not significantly associated with reductions in respiratory and gastrointestinal diagnoses, although point estimates were lower for respiratory diagnoses and slightly higher for gastrointestinal diagnoses. Azithromycin has previously been shown to have gastrointestinal side effects, which may have contributed to the observed gastrointestinal diagnoses.^19^

The present analysis has multiple limitations. Only data from a subset of communities from the Niger site of the MORDOR trial were used, and the results may not be generalizable elsewhere. Extensive information on individual children in the study was not obtained.^20^ Only the year after the first dose of azithromycin was examined. It is possible that azithromycin would yield different or more exaggerated results with additional doses. Some villages farther away from the health center had no recorded clinic visits, which limits the information gained from their inclusion. Individuals who did not visit the clinic were not captured in the present study, providing an incomplete picture of the effect of azithromycin on specific infections. Other sources of healthcare could be monitored in future studies. The clinic records in the current study did not allow for monitoring antibiotic resistance, like other study designs that involve the mass distribution of azithromycin.^21^^–^^23^

## CONCLUSION

Overall, treating communities with azithromycin in a high-mortality setting resulted in a reduction in the number of clinic visits in the present study. Clinic antibiotic prescriptions were lower in the communities that had received azithromycin. Amoxicillin was the most commonly distributed antibiotic in the study area. Therefore, decreasing clinic-prescribed amoxicillin may decrease resistance pressure to β-lactam antibiotics. Mass azithromycin distributions may have benefits beyond eliminating trachoma and reducing childhood mortality. Monitoring at the clinic level is an economically favorable method for evaluating community interventions, and it can be scaled up for far larger studies.

## Supplemental Materials

10.4269/ajtmh.25-0462 Supplemental Materials
