# Supplementary material for: Mass Azithromycin Distributions and Childhood Clinic Visits in Niger: A Community-Randomized Trial
Source: Am J Trop Med Hyg. 2025 Oct 23;113(6):1419–22. doi: 10.4269/ajtmh.25-0462 (PMC12676582; doi:10.4269/ajtmh.25-0462)
Supplement: Supplemental Materials [file tpmd250462.SD1.pdf]

Supplemental Figure 1

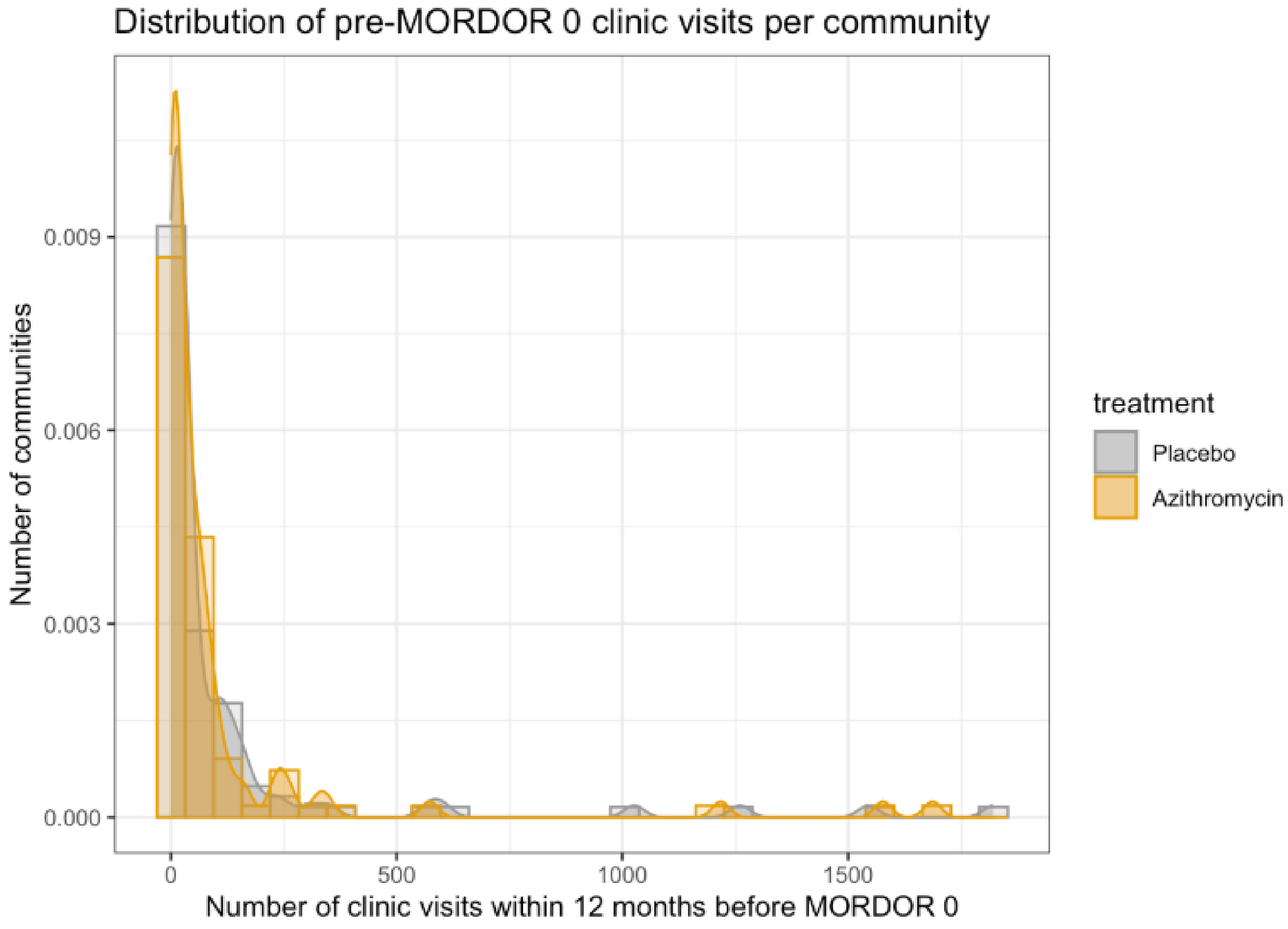

Figure 1. Number of clinic visits per community by arm within 12 months before the first distribution of azithromycin.
